# Continuous Production of Biorenewable, Polymer‐Grade Lactone Monomers through Sn‐β‐Catalyzed Baeyer–Villiger Oxidation with H_2_O_2_

**DOI:** 10.1002/cssc.201701298

**Published:** 2017-09-07

**Authors:** Keiko Yakabi, Thibault Mathieux, Kirstie Milne, Eva M. López‐Vidal, Antoine Buchard, Ceri Hammond

**Affiliations:** ^1^ Cardiff Catalysis Institute Cardiff University, Main Building Park Place Cardiff CF10 3AT UK; ^2^ Centre for Sustainable Chemical Technologies (CSCT) Department of Chemistry University of Bath Bath BA2 7AY UK

**Keywords:** heterogeneous catalysis, Lewis acids, monomers, oxidation, zeolites

## Abstract

The Baeyer–Villiger oxidation is a key transformation for sustainable chemical synthesis, especially when H_2_O_2_ and solid materials are employed as oxidant and catalyst, respectively. 4‐substituted cycloketones, which are readily available from renewables, present excellent platforms for Baeyer–Villiger upgrading. Such substrates exhibit substantially higher levels of activity and produce lactones at higher levels of lactone selectivity at all values of substrate conversion, relative to non‐substituted cyclohexanone. For 4‐isopropyl cyclohexanone, which is readily available from β‐pinene, continuous upgrading was evaluated in a plug‐flow reactor. Excellent selectivity (85 % at 65 % conversion), stability, and productivity were observed over 56 h, with over 1000 turnovers (mol product per mol Sn) being achieved with no loss of activity. A maximum space–time yield that was almost twice that for non‐substituted cyclohexanone was also obtained for this substrate [1173 vs. 607 g(product) kg(catalyst)^−1^ cm^−3^ h^−1^]. The lactone produced is also shown to be of suitable quality for ring opening polymerization. In addition to demonstrating the viability of the Sn‐β/H_2_O_2_ system to produce renewable lactone monomers suitable for polymer applications, the substituted alkyl cyclohexanones studied also help to elucidate steric, electronic, and thermodynamic elements of this transformation in greater detail than previously achieved.

## Introduction

The use of commodity polymers, such as polyesters and polyolefins, is widespread in our society. Their range of applications is highly varied, and includes the textile, agricultural, automotive, and healthcare industries, and applications in storage and packaging, among several other fields. Such materials are highly desired as they are light, strong, and cheap. In light of this, over 300 Mt a^−1^ of plastics are produced globally.^1^

At present, almost all polymeric materials are based on fossil feedstock. Owing to the finite nature of fossil feedstock and its negative environmental impact, the use of sustainable resources for polymeric materials has gathered increasing attention.^2^ Although the development of poly(lactic acid), a biodegradable polymeric material produced from renewable lactic acid, represents a significant breakthrough, such bio‐based and biodegradable polymers currently only account for <1 % of polymers produced on an annual basis.^3^ Moreover, their utilization is strongly limited by their cost and intrinsic properties, particularly their lack of functionalization. Accordingly, the development of new bio‐based polymers with high degrees of functionality represents a significant challenge.

**Scheme 1:**
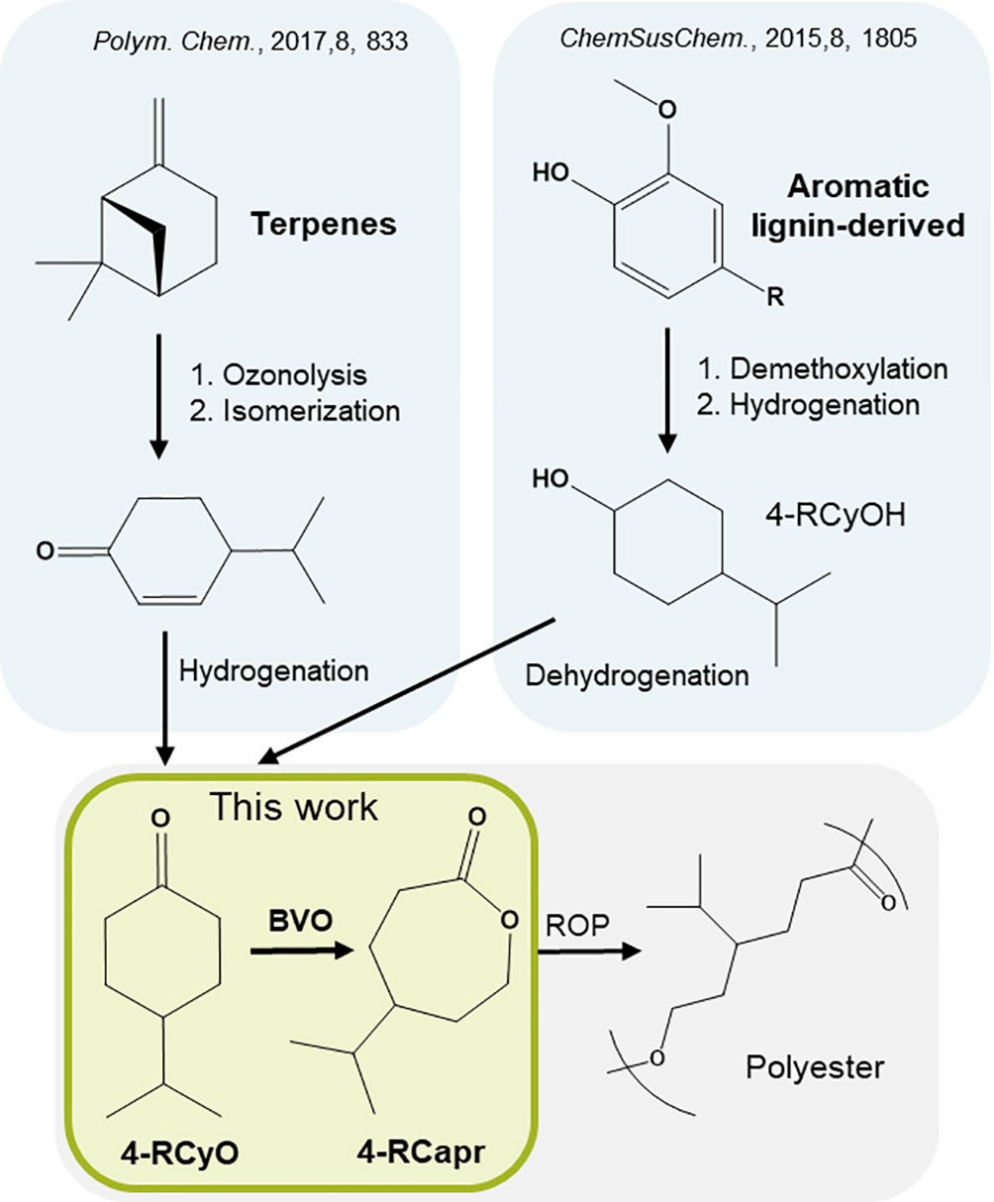
Strategies for terpene and lignin valorization to give renewable polyesters.

The first step in tackling this great challenge involves the development of new bio‐based monomer structures, preferably with tailored physical properties. Substituted ϵ‐caprolactones (R‐Capr) are valuable monomer building blocks for a variety of versatile functional polyesters, as well as elastomers by block copolymerization with lactic acid,^4^ with potential pharmaceutical and medical applications, owing to their biocompatibility, and favorable mechanical and chemical properties.^5^ A broad palette of biomass sources is suitable for renewable monomer formation. For example, naturally available terpenes and terpenoids, such as β‐pinene or d‐limonene, have been reported as precursors for substituted ϵ‐caprolactones (Scheme 1).^6^ Lignin, the largest renewable feedstock for aromatic compounds, has also been revealed to be a promising source for substituted ϵ‐caprolactones (Scheme 1).^7^ Despite their differing pathways, both routes share a common final step, where a substituted cyclohexanone (R‐CyO) is converted by Baeyer–Villiger oxidation (BVO) into the corresponding lactone monomer. In such sustainable processes in which multiple steps are required, optimization of each step is crucial to minimize costs and maximize productivity and selectivity. The study of the final steps, that is, the BVO of substituted ketones and polymerization of the resulting lactones, represents the focal point of this work.

The BVO of ketones to lactones and esters has been widely studied over the past 100 years.^8^ With the aim of increasing the sustainability of this reaction, traditionally used peracid‐based oxidants, such as peracetic acid,^9^ have recently been replaced by hydrogen peroxide (H_2_O_2_), a green oxidant with several favorable properties.^10^ Among several suitable heterogeneous catalysts, the Lewis acidic silicate Sn‐β has been shown to be an excellent catalytic material for this reaction, suitably polarizing the carbonyl group and making it more susceptible to nucleophilic attack. Since the first report of its activity for this reaction,^11^ this catalytic system has received increasing amounts of attention. Although several studies have focused on this system for one particular substrate (cyclohexanone, a precursor to ϵ‐caprolactone),^12^ the general applicability and scalability of the system remain relatively unexplored. Moreover, control of the lactone selectivity, particularly at high levels of conversion, remains a formidable challenge, owing to unavoidable ring opening hydrolysis resulting in hydroxyacid formation.^13^

Motivated by these challenges and the possibility of employing this system as a sustainable step towards the catalytic generation of renewable lactones from biomass sources, the focal points of this work are as follows: 1) To identify the general applicability of the Sn‐β/H_2_O_2_ BVO system, particularly in terms of substrate scope; 2) to further evaluate its activity, selectivity, and stability in the continuous regime, particularly for renewable‐based ketones; 3) to probe the suitability of the lactones produced during catalysis for downstream applications.

## Results and Discussion

### Catalyst synthesis and characterization

In line with our recent studies, including those focused on the BVO of cyclic ketones, microporous Sn‐β was prepared by solid‐state incorporation (SSI).12a A Sn loading of 2 wt % was employed, as this loading results in a material with a uniform distribution of isolated Sn^IV^ species, with negligible amounts of tin oxides (SnO_*x*_) being present.12a Where appropriate, hierarchical Sn‐β (Sn‐β‐H) was also synthesized. This material possesses a combination of micro‐ and mesopores, resulting in improved molecular transport and greater substrate accessibility.^14^ According to our optimized synthesis protocols, Sn‐β and Sn‐β‐H catalysts were prepared from commercially available Al‐β (SiO_2_/Al_2_O_3_=38), following dealumination in HNO_3_ or desilication in NaOH and dealumination, respectively.

Both materials were extensively characterized in recent studies,^15^ and for brevity the spectroscopic data will not be reproduced in the main text. Briefly, the post‐synthetic treatments employed (dealumination, or desilication followed by dealumination for Sn‐β and Sn‐β‐H, respectively) did not change the parental zeolite beta structure (see the Supporting Information, Figures S1 and S2) and Sn^IV^ was successfully incorporated in the zeolite framework almost exclusively for both materials as evidenced by ^119^Sn magic‐angle spinning (MAS) NMR and diffuse reflectance infrared Fourier transform spectroscopy (DRIFTS) studies using CD_3_CN (Figures S3 and S4).

### Extending the substrate scope: Effect of 2‐ and 4‐substituents on catalytic activity

**Scheme 2:**
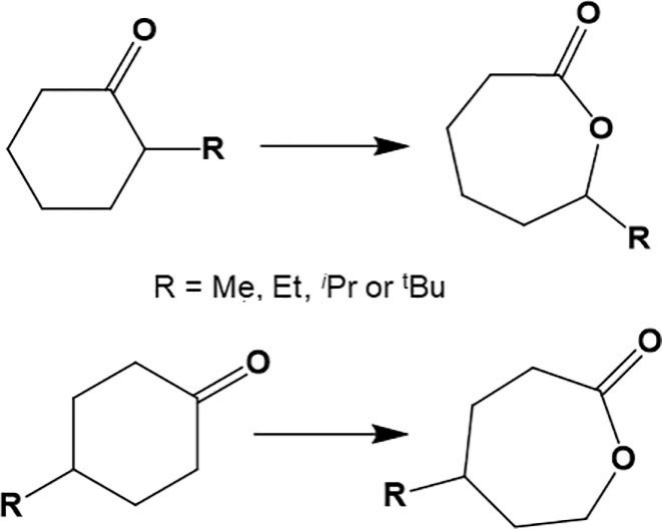
BVO of 2‐R and 4‐R‐substituted cyclic ketones.

To evaluate the general applicability of the Sn‐β/H_2_O_2_ BVO system beyond cyclohexanone, various substituted cyclohexanones were explored as substrates. Understanding the effect of substituents at different positions (2‐ and 4‐) is of great importance in order to anticipate the viability of the system to convert more complex substrates, such as terpene‐derived ketones such as 4‐isopropylcyclohexanone, into renewable lactones (Scheme 2). With this aim, we first investigated the impact of increasing the size of substituent for alkylcyclohexanones (Me, Et, *i*Pr, or *t*Bu) substituted at position 2 (2‐R‐CyO) and 4 (4‐R‐CyO). All substrates were evaluated for BVO under our standard reaction conditions (internal solution temperature 100 °C, 0.33 m substrate in 1,4‐dioxane, 1 mol % of catalyst, H_2_O_2_/ketone ratio of 1.5) and their intrinsic activity profiles compared to unsubstituted CyO.^13^

**Figure 1:**
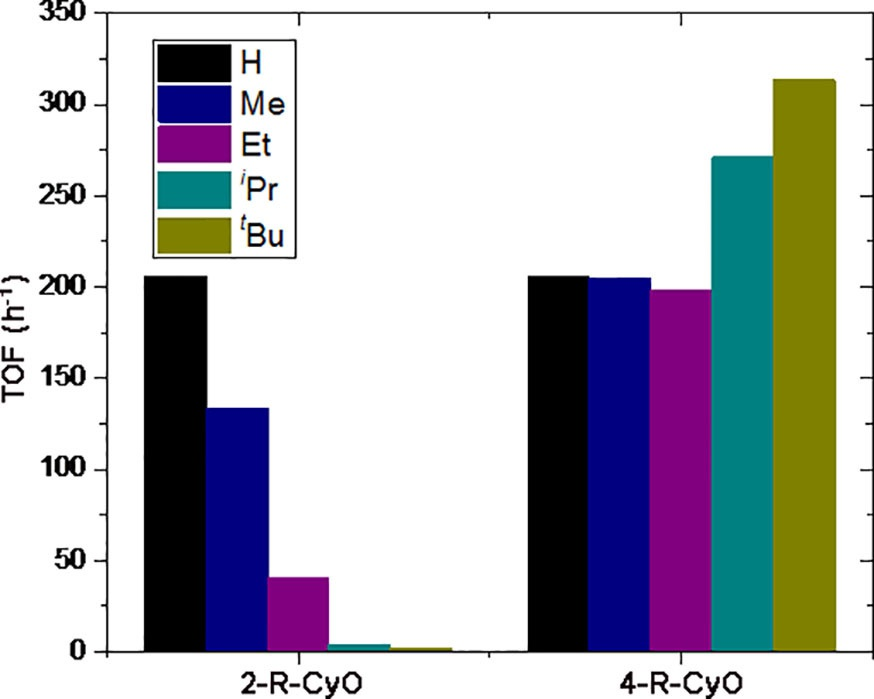
Initial TOF (10 min) obtained for the two series of BVO reactions: 2‐R‐CyO (left) and 4‐R‐CyO (right). TOF calculated as moles of R‐CyO converted per mole Sn per unit time. Reaction conditions: 0.33 m R‐CyO in 1,4‐dioxane (10 mL), H_2_O_2_/R‐CyO=1.5:1, 1 mol % Sn at 100 °C.

Despite the similar chemical nature of the substrates tested, large differences in reactivity within each series, and between both series, were observed (Figure 1). For 2‐R‐CyO, catalytic activity was found to decrease with increasing size of R, decreasing from an initial turnover frequency (TOF) of ±200 h^−1^ for CyO, to <10 h^−1^ when the bulkiest substrates (*i*Pr and above) were screened (Figure 1). We note that the TOF was calculated over the first 10 minutes of reaction as mol ketone converted per mol Sn per unit time. In contrast, excellent reactivity was observed for 4‐R‐CyO at all sizes of substituent. Indeed, the initial TOF increased by some 50 % across the series, from ±200 h^−1^ for CyO to >300 h^−1^ for 4‐*t*Bu‐CyO.

Density functional theory (DFT) studies (Table S1) indicated that while the BVO reaction of cyclohexanones into lactones is strongly thermodynamically favored, alkyl substitution does not influence the overall thermodynamics of the reaction. Hence, kinetic and/or steric factors must account for the observed changes in reactivity. Although the position and identity of the substituent will evidently impact the kinetic diameter of each substrate, it is unlikely that overall steric bulk and, hence, transport limitations account for the reactivity trends within, and between, each series. This was confirmed by the observation that hierarchical Sn‐β, which also possesses mesopores <8 nm in diameter and has been shown to catalyze the conversion of 12‐membered cycloketones,15b gave rise to the same trends in relative reactivity (Figures S5–S7). Clearly, more localized effects are present.

**Scheme 3:**
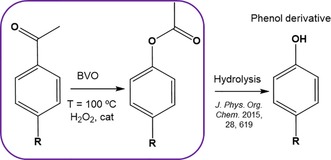
BVO of 4‐R‐AcetoPhO, including potential downstream valorization of the resulting R‐phenyl acetates.

Although not truly conjugated, both electronic and localized steric effects could contribute to the decrease in activity within the 2‐R‐CyO series at increasing size of R. To investigate possible electronic contributions to the BVO reaction of our substrates of study, we selected a range of ρ‐substituted acetophenones (4‐R‐AcetoPhO) as model substrates. Such substrates possess an electronically delocalized phenone ring that can be activated or deactivated by the *para* substituent. This can enhance or decrease its general activity for the BVO reaction depending on the potential electronic contributions present. In addition to providing a system by which potential electronic effects can be elucidated, the R‐phenyl acetates generated by BVO are an attractive source for phenol derivative formation by ester hydrolysis (Scheme 3).^16^

The effect of various electron‐donating (Hammett constant value σ<1) and electron‐withdrawing substituents (σ>1), were studied under standard BVO conditions with microporous Sn‐β as catalyst. Considering the most widely accepted reaction mechanism, based on a two‐step reaction first involving nucleophilic attack of the oxidant to the carbonyl group forming a Criegge adduct, and subsequent migration resulting in the BVO product,^17^ we hypothesized that a decrease of the BVO rate should be observed when electron‐donating R groups are present, due to lower electrophilicity at the carbonyl carbon atom. However, detailed kinetic analysis revealed that the opposite trend for all 4‐R‐AcetoPhO substrates tested occurred. Electron‐donating R groups (−NH_2_, −OAlkyl and ‐Alkyl) enhanced activity, whereas electron‐withdrawing R groups (−Cl and −NO_2_) decreased the rate substantially. For instance, the presence of −NH_2_, the most electron‐donating substituent tested, improved conversion of acetophenone from 8 % to 60 % after 6 h of reaction. In contrast, −NO_2_, the most electron‐withdrawing R group, deactivated the substrate completely (Figure S8). A typical Hammett plot further supported the observed electronic effect. Although the trend obtained was not completely linear, in general terms, higher electron‐donating ability resulted in higher initial rates of BVO with respect to acetophenone.

**Figure 2:**
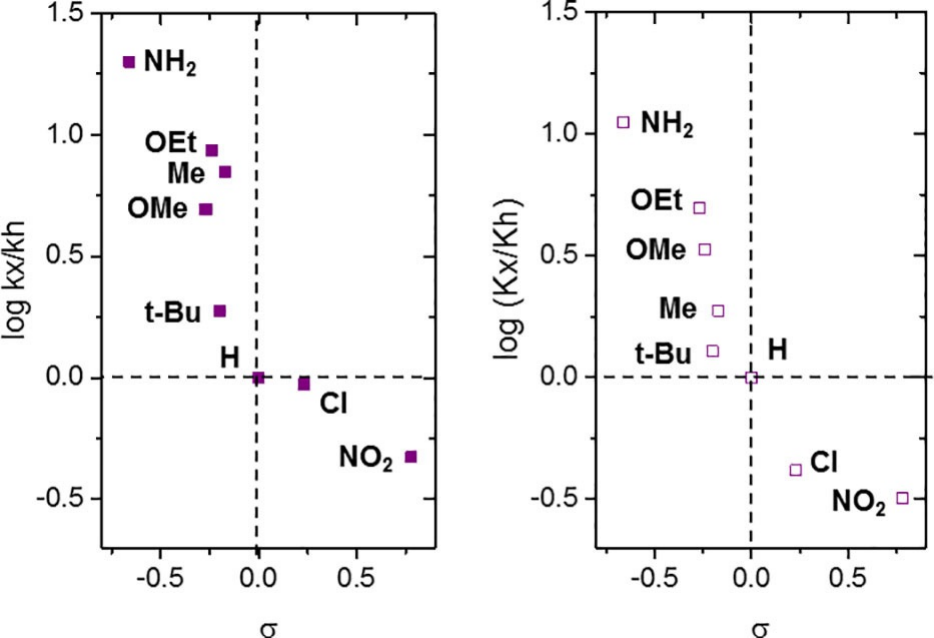
Hammett plot obtained from BVO of 4‐R‐AcetoPhO with Sn‐β (left) and Sn‐β‐H (right). Reaction conditions: 0.33 m 4‐R‐AcetoPhO in 1,4‐dioxane (10 mL), H_2_O_2_/4‐R‐AcetoPhO=1.5:1, 1 mol % Sn at 100 °C.

The 4‐R‐AcetoPhO series was also screened with Sn‐β‐H as catalyst, as additional mesoporosity may decrease potential transport contributions for the bulkiest substrates. As with Sn‐β, however, electron‐donating R enhanced the catalytic performance of the BVO reaction with respect to acetophenone (Figure S9). The Hammett plot obtained from initial rates when using Sn‐β‐H also showed a similar trend to conventional Sn‐β, but with slightly improved linearity (Figure 2).

Although unexpected based on the hypothesized reaction mechanism, the Hammett correlation obtained agrees with previous studies of classical BVO chemistry with peracid‐based oxidants.^18^ In said systems, the presence of an electron‐donating group in the *para* position results in two opposite effects: while it decreases the electrophilicity of the carbonyl group, it also increases the basicity of the carbonyl oxygen, facilitating its protonation. Once protonated, the electrophilicity of the carbonyl group is increased dramatically, thus favoring attack by the oxidant. Despite the differences with our system, the same principle can be invoked here; given that the Lewis acid Sn sites of the catalyst need to coordinate to, and subsequently polarize, the C=O bond of the substrate to promote nucleophilic attack of H_2_O_2_, it may be that electron‐donating R groups enhance coordination of the substrate, facilitating attack by the oxidant.

Regardless of the mechanistic consequences, the acetophenone results clearly demonstrate that potential electronic effects cannot account for the decrease in activity with increasing size of R in 2‐R‐CyO, since an increase in the BVO rate would be expected across the series based purely on electronic factors. In view of this, it is likely that the increasing bulk of R in the second position hinders the reaction through steric contributions. Indeed, it can be anticipated that coordination of the C=O group to the Sn sites within the zeolite framework will be hindered greatly by the presence of a bulky R group in the second position. However, this effect is likely to be very dependent upon the precise speciation, composition, and properties of the catalyst, particularly the precise T‐site location of the Sn atoms, and is therefore likely to be strongly influenced by the preparation methodology, especially because Sn‐β samples prepared by hydrothermal synthesis exhibit different trends.7b

### Extending the substrate scope: Effect of 2‐ and 4‐substituents on reaction selectivity

In addition to increased activity, substitution of the cyclohexane ring results in major changes in reaction selectivity, especially for the 4‐R‐CyO series. In our previous studies focused on the BVO of CyO with Sn‐β/H_2_O_2_,^13^ we demonstrated that a decrease in lactone selectivity occurs as the level of ketone conversion increases beyond approximately 60 %. This occurs through hydrolysis of the lactone product, resulting in the formation of the corresponding hydroxyacid [6‐hydroxyhexanoic acid (6‐HHA) in the case of CyO]. In addition to decreasing selectivity, the hydroxyacid product also deactivates the catalyst by poisoning. Unfortunately, our previous work revealed the consecutive ring opening reaction to be unavoidable for CyO, as it is also catalyzed by Sn‐β. The unavoidable formation of the ring‐opened hydroxyacid is currently a major limitation of the Sn‐β/H_2_O_2_ system for ϵ‐caprolactone formation.

**Figure 3:**
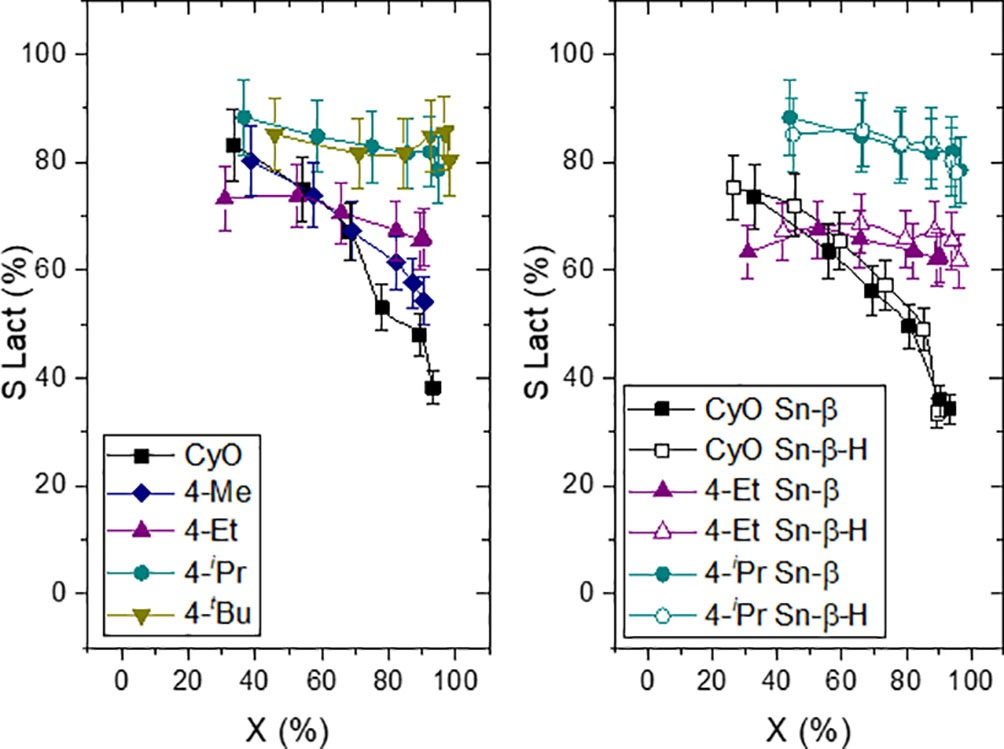
Selectivity to 4‐R‐lactone (*S*‐Lact) as a function of 4‐R‐CyO conversion with Sn‐β (left) and selectivity to lactone obtained with Sn‐β and Sn‐β‐H as function of conversion of 4‐R‐CyO (right).

To investigate the impact of alkyl substitution on reaction selectivity, we monitored lactone selectivity as a function of substrate conversion for a variety of substituted ketone substrates (Figure 3, left). Given their rapid rates of reaction, we primarily focused upon 4‐substituted ketones (4‐R‐CyO). The lactone selectivity obtained for each substrate is higher at all overlapping levels of conversion, following substitution at the 4 position. The improved selectivity is especially notable at high levels of conversion at which the consecutive hydrolysis reaction occurs more readily. Indeed, lactone selectivity at particular levels of conversion increases as the size of the 4‐R substituent increases. For example, whereas the lactone selectivity decreased to <50 % at a conversion of 80 % for CyO, lactone selectivity for both 4‐*i*Pr‐CyO and 4‐*t*Bu‐CyO remained above 80 % throughout the reaction, and became independent of the extent of conversion. Quantification of all reaction products confirmed that ring‐opening hydrolysis accounts for the remaining carbon balance for each substrate (Figures S10–S12). Given the negative role of hydroxyacids in the Sn‐β/H_2_O_2_ system (see above), the outstanding results obtained for 4‐R‐CyO, especially in terms of high lactone selectivity at high levels of conversion, points towards them as being especially promising substrates for renewable lactone formation.

Given that Sn‐β also catalyzes the hydrolysis of the lactone product, we considered the possibility that pore size limitations experienced by the bulkier 4‐R‐Capr products may inhibit their access to Sn sites following BVO, thus decreasing consecutive ring opening. To evaluate this hypothesis, the conversion‐selectivity profiles obtained for the BVO of CyO, 4‐Eth‐CyO and 4‐*i*Pr‐CyO over Sn‐β‐H and Sn‐β were compared (Figure 3, right). If confinement and pore size limitations were key to inhibiting the interaction of the product lactone and the Sn sites, hydrolysis of the lactone should be more dramatic when using the hierarchical catalyst. Nevertheless, no significant differences in terms of activity or selectivity were observed for the three substrates when using Sn‐β‐H, and the selectivity trends towards substituted lactones by using both materials were maintained, that is, higher lactone selectivity was still observed even when mesopores <8 nm are present.

**Figure 4:**
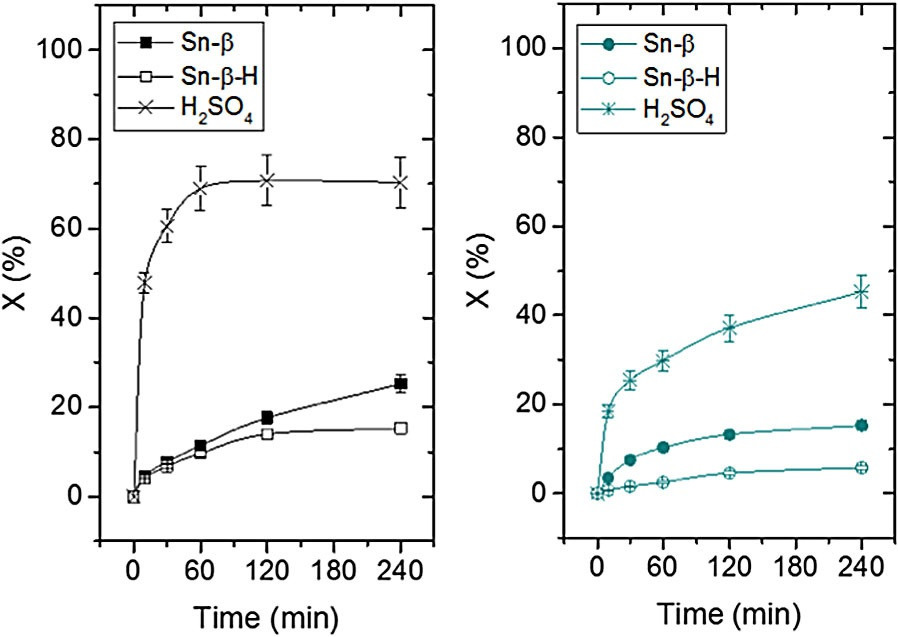
Caprolactone (left) and 4‐*i*Pr‐Capr (right) hydrolysis performed with Sn‐β, Sn‐β‐H, and H_2_SO_4_.^[a]^ Reaction conditions (unless otherwise stated): 0.2 m lactone in 1,4‐dioxane (10 mL), H_2_O (0.15 mL), 1 mol % Sn, 100 °C. [a] H_2_SO_4_ equivalent to usual 1 mol % Sn used.

To verify that the substituted lactone products are intrinsically more resistant to ring opening and that pore‐size limitations do not decrease the extent of hydrolysis through steric effects, a study of the hydrolysis of isolated 4‐*i*Pr‐Capr was performed (Figure 4, right). Three catalysts (Sn‐β, Sn‐β‐H, and H_2_SO_4_) were screened for hydrolysis activity using the same amount of water present in a typical BVO reaction from aqueous H_2_O_2_. If pore‐size limitations were responsible for the decreased levels of hydrolysis occurring to 4‐*i*Pr‐Capr, then one would expect hydrolysis to occur more readily for Sn‐β‐H, which is hierarchical in nature and possesses a combination of micro‐ and mesopores. Although both heterogeneous catalysts are less active than H_2_SO_4_, it is clear that the co‐presence of mesopores is not beneficial to the rate of hydrolysis as the rate of hydrolysis is similar, if not lower, when hierarchical Sn‐β‐H is employed as catalyst. It is also notable that the rate of hydrolysis of 4‐*i*Pr‐Capr is much lower than that of Capr even when using homogeneous catalysts, confirming that the substituted lactones are intrinsically more resistant to hydrolysis. Further verification of this was achieved through a thermodynamic study of the hydrolysis reaction with DFT (Table S2). These calculations were consistent with the experimental data, indicating that increasing substitution at the 4‐position makes hydrolysis more thermodynamically disfavored, despite the substitution being relatively remote from the lactone function. We note here, therefore, that the hydrolysis reaction is clearly disfavored for substituted lactones for both thermodynamic (DFT) and kinetic (experimental) reasons.

### Extending the substrate scope: Intensification of 4‐*i*Pr‐CyO BVO in continuous flow

Given that 4‐substituted ketones, such as 4‐*i*Pr‐CyO, are more active for BVO, are more resistant to undesirable hydrolysis, and are also available from renewable resources such as β‐pinene, they appear to be especially promising substrates for BVO upgrading. Accordingly, intensification of the BVO of 4‐*i*Pr‐CyO was investigated in the continuous regime with Sn‐β as catalyst. Performance, in terms of stability and productivity (space–time‐yield) was compared to that obtained during the BVO of CyO.

After a short induction period, during which both activity and selectivity increase, both systems (CyO and 4‐*i*Pr‐CyO) reach a steady‐state level of performance over the reaction period explored (56 h), with little or no loss of activity being observed. Selectivity continues to increase during the initial cycle, reaching a maximum of 85 % after 50 h of operation. During this period, >1000 turnovers (mol product per mol. Sn) are achieved for 4‐*i*Pr‐CyO, equivalent to >10 batch reactions under typical conditions. This clearly demonstrates the stability of the Sn‐β/H_2_O_2_ catalytic system for the BVO of more functionalized starting substrates.

**Figure 5:**
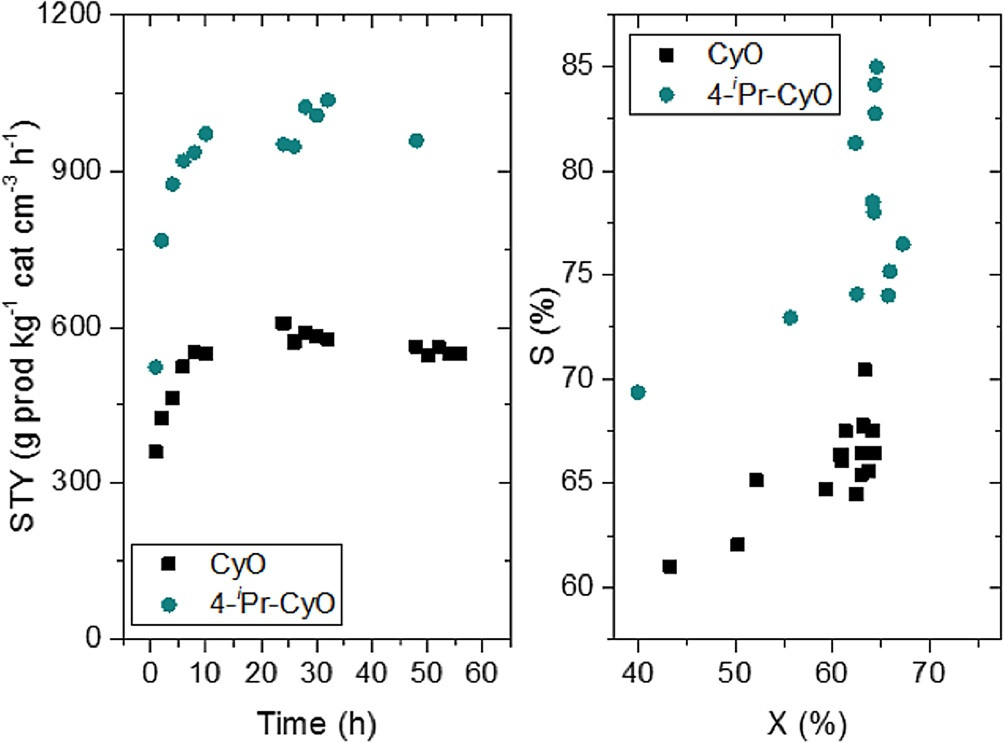
Left) Space–time yield obtained for BVO of CyO and 4‐*i*Pr‐CyO in continuous operation. Space–time yield calculated as grams of lactone produced per kg of catalyst per unit reactor volume per unit time. Right) Selectivity to lactone as a function of ketone conversion during continuous operation. Reaction conditions: Reactant feed of 0.33 m cyclohexanone and 0.5 m H_2_O_2_ in 1,4‐dioxane with a contact time of 5.5 min under a pressure of 10 bar, at 100 °C.

Although both systems exhibit comparable levels of stability, it is notable that under identical conditions, the continuous BVO of 4‐*i*Pr‐CyO results in higher levels of activity (conversion) and selectivity (Figure 5, right). In addition to corroborating the batch results (see above), the increased levels of activity and selectivity combine to yield substantial improvements in space–time yield over the reaction period. Indeed, the maximum space–time‐yield obtained during the BVO of 4‐*i*Pr‐CyO is almost two‐times higher than that obtained during the BVO of CyO (1173 vs. 607 g kg^−1^ cm^−3^ h^−1^, respectively; Figure 5, left). Thus, in addition to being a renewable and sustainable source of carbon for chemical production, 4‐substituted ketones such as 4‐*i*Pr‐CyO are more active, selective, and productive substrates for BVO chemistry.

### Probing lactone quality: Polymerization studies

To demonstrate the viability of the lactone produced through this BVO process for polymer formation, ring‐opening polymerization of 4‐*i*Pr‐Capr, isolated by simple column chromatography, was performed under industrially relevant melt conditions at 110 °C, using triazabicyclodecene (TBD) as catalyst (1 mol %) and 4‐methylbenzyl alcohol (1 mol %) as initiator. Previously, only metal‐catalyzed polymerization has been reported for such substrates. After a period of 24 h, 30 % conversion was observed. The produced polymer possessed a number‐average molecular weight (*M*_n_) of 1384 g mol^−1^, and dispersity of 1.27, clearly demonstrating that the lactone produced through BVO is suitable for polymer applications even without excessive pretreatments. Future studies will further explore the potential of monomers produced through this BVO process for polymer formation.

## Conclusions

4‐substituted cycloketones, such as 4‐isopropyl cyclohexanone (4‐*i*Pr‐CyO), are readily available from a variety of renewable resources, including terpenes and lignin. Herein, we show such substrates are especially favorable as platforms for Baeyer–Villiger oxidation, which is a key step in the production of renewable lactone monomers. In addition to being renewable, we show that such substrates are also more active, more selective, and more productive substrates for BVO relative to unsubstituted cycloketones, such as cyclohexanone. Optimal performance was observed for 4‐*i*Pr‐CyO. Successful intensification of its BVO in the continuous regime was demonstrated, with excellent levels of stability, productivity and selectivity observed over >1000 substrate turnovers. In this case, the maximum space–time yield was almost twice that obtained during BVO of cyclohexanone, reaching 1173 g(product) kg(catalyst)^−1^ cm^−3^ h^−1^, at a lactone selectivity of 85 %. Various electronic, steric, and thermodynamic effects are shown to contribute to the outstanding reactivity of such substituted cyclic ketones in the Baeyer–Villiger oxidation. The lactone produced by this continuous experiment is also shown to be suitable for downstream polymerization, being effectively polymerized at mild, metal‐free conditions. The outstanding results obtained in both batch and continuous mode clearly demonstrates the successful broadening of the substrate scope of the Sn‐β/H_2_O_2_ BVO system. Moreover, they open a route towards the sustainable production of renewable monomers through the BVO of sustainably derived ketones.

## Experimental Section

### Catalyst synthesis

Sn‐β catalyst was prepared as follows: Zeolite Al‐β (Zeolyst, NH_4_‐form, SiO_2_/Al_2_O_3_=38) was dealuminated by treatment in HNO_3_ solution (13 m HNO_3_, 100 °C, 20 h, 20 mL g^−1^ zeolite). The dealuminated powder was washed with water and dried overnight at 110 °C. Solid‐state stannation was performed by grinding the appropriate amount of tin(II) acetate with the necessary amount of dealuminated zeolite for 10 minutes in a pestle and mortar, prior to heat treatment in a combustion furnace (Carbolite MTF12/38/400). A two stage heat treatment was employed: to 550 °C (10 °C min^−1^ ramp rate) first in a flow of N_2_ (3 h not including ramp period) and subsequently air (3 h) for a total of 6 h plus heating and cooling periods. Gas flow rates of 60 mL min^−1^ were employed at all times. The sample was held horizontally in an alumina combustion boat (10 mL capacity) and a quartz tube was used to seal the sample environment.

Hierarchical Sn‐β (Sn‐β‐H) was prepared as follows: Commercial Al‐β Zeolite (CP814E, NH_4_‐form, SiO_2_/Al_2_O_3_=38, Zeolyst International) was first converted into the protonic form by calcination in static air at 550 °C (5 °C min^−1^) for 5 h. The H‐form zeolite was subsequently suspended in an aqueous solution of NaOH (0.2 m, 318 K, 0.5 h, 30 mL g^−1^ zeolite) to obtain a desilicated zeolite β (deSi‐β). The reaction was stopped immediately by cooling the container in an ice bath. The remaining solid product was centrifuged, thoroughly washed with deionized water until neutral pH was attained, and finally dried at 373 K overnight. Subsequently, deSi‐β was dealuminated following the same procedure described above. After filtering and drying the sample, the sample was reconverted into NH_4_‐form by ion exchange in a solution of NH_4_NO_3_ (1 m, 2 h, 85 °C, 30 mL solution per gram of catalyst). Solid‐state incorporation of Sn was performed as described above.

### Kinetic evaluation and analytical methods

Batch BVO reactions were performed in a 100 mL round‐bottom flask equipped with a reflux condenser. Reaction temperature was controlled by immersion in a silicone oil bath. The vessel was charged with a 0.33 m solution of substrate in 1,4‐dioxane (10 mL), which also contained an internal standard (biphenyl, 0.01 m) and the appropriate amount of catalyst. The vessel was subsequently heated to the desired temperature (100 °C internal temperature). The reaction was initiated by addition of an appropriate amount of H_2_O_2_ (50 wt % in aqueous solution), typically corresponding to a H_2_O_2_/ketone ratio of 1.5. The solution was stirred at ±750 rpm with an oval magnetic stirrer bar. Aliquots of reaction solution were taken periodically for analysis, and were centrifuged prior to injection into a GC (Agilent 7820, 25 m CP‐Wax 52 CB). Reactants were quantified against a biphenyl internal standard. Quantification of the byproducts by ^1^H NMR spectroscopy was achieved by calibrating a capillary containing a known amount of tetramethylsilane (TMS; s, *δ*=0.0 ppm) against known standards, and placing this capillary within the NMR tubes of various time online samples.

R‐Caprolactones were obtained as colorless oils from the BVO reactant solutions and separate by column chromatography (silica gel, hexane/ethyl acetate=3:1 *v*/*v*). Batch R‐caprolactone hydrolysis reactions were performed following the same procedure and reaction conditions as used for the BVO reaction, although R‐Cyclohexanones were replaced with R‐caprolactone and oxidant was replaced with the equivalent water amount added.

Continuous BVO reactions were performed in a home‐made plug‐flow stainless steel tubular reactor. Reactant delivery (0.33 m R‐CyO in 1,4‐dioxane, H_2_O_2_/ketone=1.5) was performed by an HPLC pump. The catalyst (0.2 g) was mixed with a diluent material (SiC, particle size=63–75 μm, 0.8 g) to prevent back mixing and to minimize pressure drop, and the bed placed in between two plugs of quartz wool. The diluted catalyst was densely packed into a 1/4 in. stainless steel tube (i.d.=4.1 mm) and a frit (0.5 μm) was placed at the end of the bed to prevent any loss of material. A contact time of 5.5 min was typically employed. The reactor temperature was controlled by immersion in a thermostatic oil bath, and pressure was controlled by means of a back‐pressure regulator (10 bar). The reaction feed was identical to that used for batch reactions. Aliquots of the BVO reaction solutions were taken periodically from a sampling valve placed after the reactor, and were analyzed in the same manner as the batch reactions.

### Polymerization

In a glovebox, 4‐*i*Pr‐Capr (0.35 g, 2.2 mmol), triazabicyclodecene (22 μL of 1 mol L^−1^ solution in anhydrous CH_2_Cl_2_), 4‐methylbenzylalcohol (22 μL of 1 mol L^−1^ solution in anhydrous CH_2_Cl_2_) were mixed and sealed in flask under argon, before transferring to an oil bath preheated at 110 °C. After the required time, the flask was opened to air, quenched with a few drops of MeOH, and the solvent removed under reduced pressure. A crude NMR spectrum was recorded to determine the conversion of monomers from the relative integrals of monomer and polymer resonances.^6^ The polymer was then washed with MeOH to remove unreacted monomer before drying under vacuum and analysis by size‐exclusion chromatography (SEC). SEC was carried out on an Agilent Technologies 1260 Infinity instrument with a flow rate of 1 mL min^−1^ at 35 °C with THF as eluent and referenced against polystyrene standards (RI).

### Computational details

All calculations were performed by using the Gaussian 09 suite of codes (revision D.01).^19^ Geometries were fully optimized without any symmetry or geometry constraints using various functional (ωB97XD,^20^ B3LYP‐D3^21^ and M06‐2X).^22^ Calculations were carried out using a temperature of 398.15 K and solvent effects in 1,4‐dioxane were considered by using a conductor‐like polarizable continuum model (CPCM).^23^ The nature of all the stationary points as minima was verified by calculations of the vibrational frequency spectrum. Free energies were calculated within the harmonic approximation for vibrational frequencies. Only the most stable conformational isomers are reported for all intermediates.

## Conflict of interest

*The authors declare no conflict of interest*.

## Supporting information

As a service to our authors and readers, this journal provides supporting information supplied by the authors. Such materials are peer reviewed and may be re‐organized for online delivery, but are not copy‐edited or typeset. Technical support issues arising from supporting information (other than missing files) should be addressed to the authors.

Supplementary
