# Supplementary material for: Continuous Production of Biorenewable, Polymer‐Grade Lactone Monomers through Sn‐β‐Catalyzed Baeyer–Villiger Oxidation with H2O2
Source: ChemSusChem. 2017 Sep 7;10(18):3652–9. doi: 10.1002/cssc.201701298 (PMC5708276; doi:10.1002/cssc.201701298)
Supplement: Supplementary file 1 — Supplementary [file CSSC-10-3652-s001.pdf]

CHEMISTRY & SUSTAINABILITY
CHEMSUSCHEM
ENERGY & MATERIALS

Supporting Information

Continuous Production of Biorenewable, Polymer-Grade Lactone Monomers through Sn-β-Catalyzed Baeyer–Villiger Oxidation with H_2O_2

Keiko Yakabi,[a] Thibault Mathieux,[a] Kirstie Milne,[a] Eva M. López-Vidal,[b] Antoine Buchard,[b] and Ceri Hammond*[a]

cssc_201701298_sm_miscellaneous_information.pdf

Extended Supplementary Information for:

Continuous production of biorenewable, polymer-grade lactone monomers through Sn-β-catalyzed Baeyer-Villiger oxidation with H_2O_2

Keiko Yakabi,[a] Thibault Mathieux,[a] Kirstie Milne,[a] Eva M. López-Vidal,[b] Antoine Buchard[b] and Ceri Hammond*[a]

[a] Cardiff Catalysis Institute, Cardiff University, Main Building, Park Place, Cardiff, CF10 3AT, UK
[b] Centre for Sustainable Chemical Technologies (CSCT), Department of Chemistry, University of Bath, Bath, BA2 7AY, UK

KEYWORDS. Heterogeneous catalysts; Lewis Acids; Monomers; Oxidation; Zeolites

hammondc4@cardiff.ac.uk

WILEY-VCH

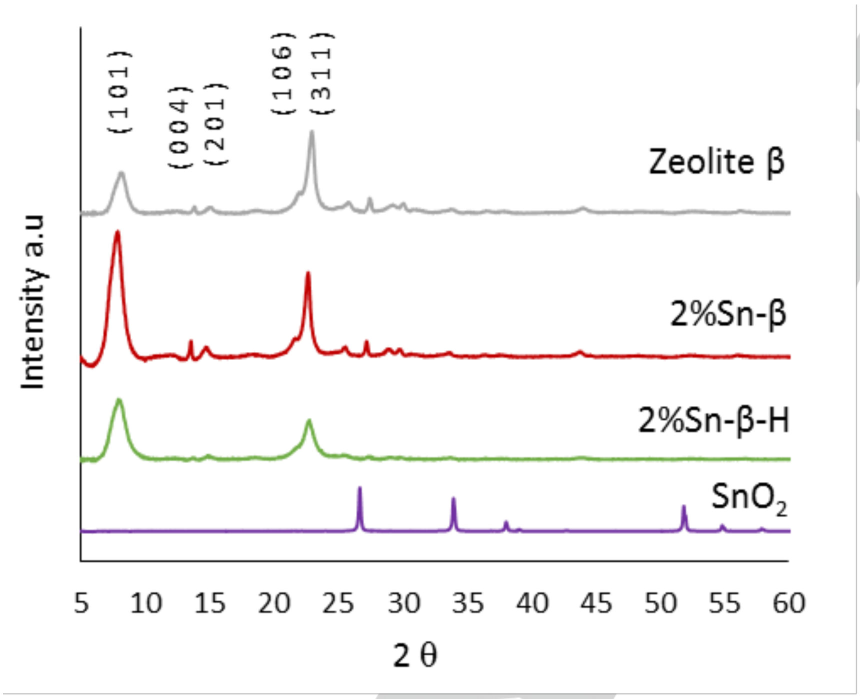

Figure S1. XRD patterns for commercial zeolite β, 2%Sn-β, 2%Sn-β-H and SnO_2. Preservation of the main peaks in both Sn-catalysts patterns, and comparable diffraction intensity with parent zeolite, indicates that the crystalline phase structure of zeolite β remained unaltered after the post-synthetic treatments and in the absence of secondary phases.

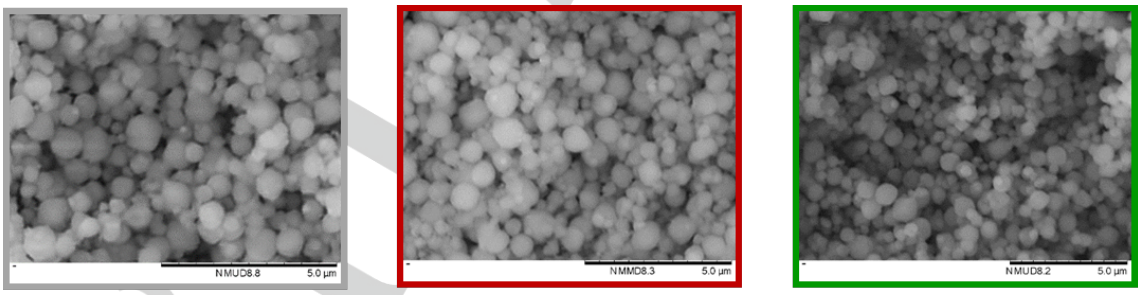

Figure S2. SEM images of commercial zeolite β (left), 2%Sn-β (centre) and 2%Sn-β-H (right). Crystals of spherical shape agglomerated and size ranged from 20 to 90 nm were maintained after postsynthetic treatments, indicating that desilication and dealumination treatments did not overly damage the zeolite crystallites.

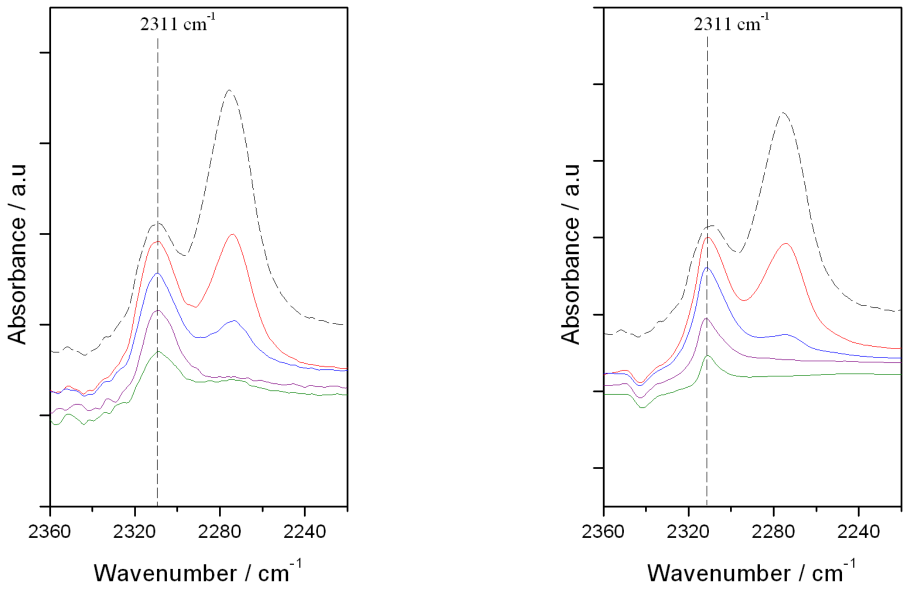

Figure S3. In situ DRIFTS spectra of (right) 2%Sn-β, and (left) 2%Sn-β-H, following (black) 10 min treatment with CD_3CN at room temperature. Various desorption temperatures (50 (red), 100 (blue), 150 (purple)) are also displayed. The first band at 2311 cm^{-1} was associated with CD_3CN coordinated onto the Sn (IV) Lewis acid sites, and demonstrated that Sn (IV) was isolated and present in the zeolite framework. The second IR band is due to weakly physisorbed acetonitrile onto the samples.

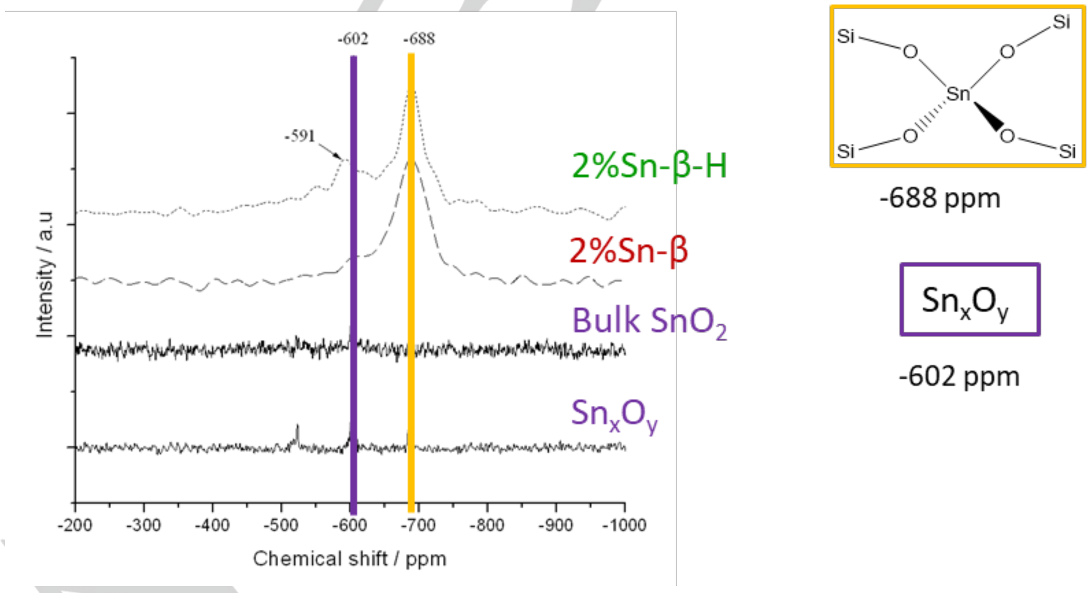

Figure S4. MAS NMR spectra of SnO_2, 10% SnO_2/deAl-β (physical mixture), 2%Sn-β and 2%Sn-β-H. Both kind of zeolites present a major resonance signal at -688 ppm that demonstrate the presence of Sn (IV) sites and their predominance among all the material. Small resonance signal at -602 ppm can be observed too, being more noticeable in 2%Sn-β-H than in 2%Sn-β, indicating the presence of some oxide species.

Thermodynamics of the Baeyer-Villiger oxidation with H_2O_2 of 4-alkyl-cyclohexanone

The increasing intrinsic reactivity of 4-R-CyO against CyO for BVO was evaluated using Density Functional Theory by calculating the thermodynamics of the reaction with H_2O_2 for each substrate. Geometry optimisation were performed at the ωB97XD level and 398.15 K, using a mixed valence triple ζ basis set 6-311++g(2d,p) for all atoms, and a conductor-like polarisable continuum model of 1,4-dioxane. The results of these calculations are summarised in Table S1. Full coordinates for all the stationary points, together with computed Gibbs free energy and vibrational frequency data, are available via the corresponding Gaussian 09 output files, stored in the digital repository: DOI: 10.6084/m9.figshare.5113126.

Table S1. DFT computed Free Gibbs energies of the oxidation of 4-R-cyclohexanone into 4-R-caprolactone[a]

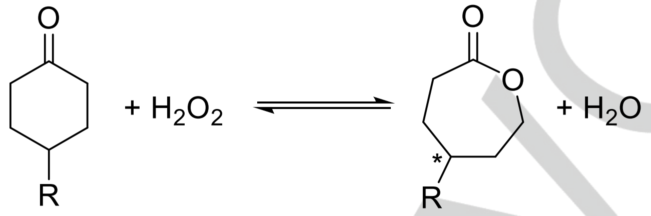

**4-R-Caprolactone**	**ΔG (kcal mol^{-1})**[b]
R = H	– 65.2
R = Me	– 65.0
R = Et	– 65.0
R= ^{i}Pr	– 64.0
R= ^{t}Bu	– 64.7

[a] Calculations were performed on the (S)- enantiomers, with the alkyl substituent in equatorial position.
[b] Protocol: ωB97XD/6-311++g(2d,p)/ 398.15K/cpcm=1,4-dioxane

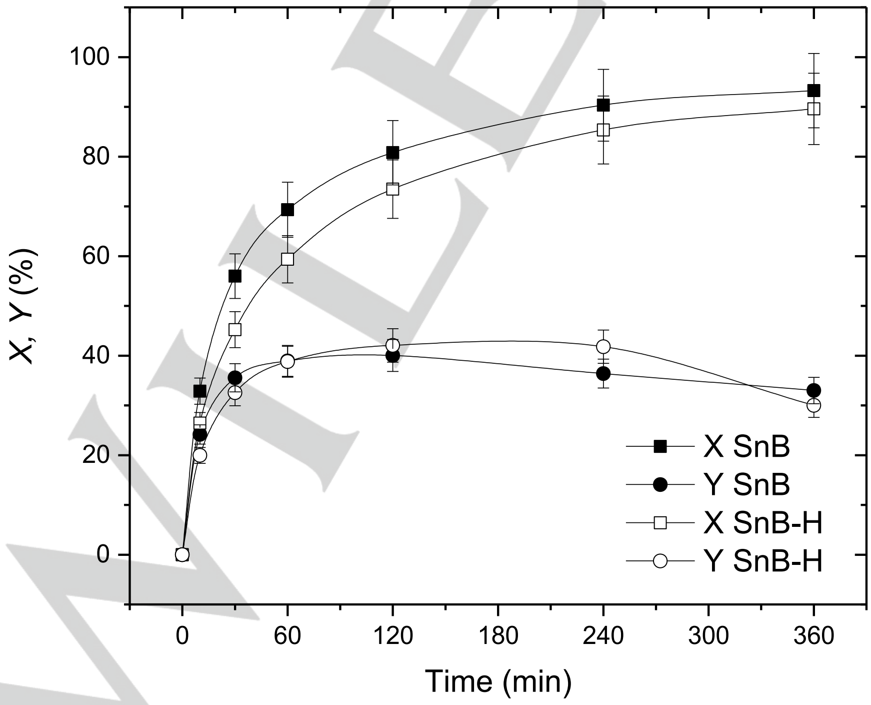

Figure S5. Time online analysis of BVO reaction of CyO with Sn-β and Sn-β-H, where X represents substrate conversion and Y represents lactone yield.

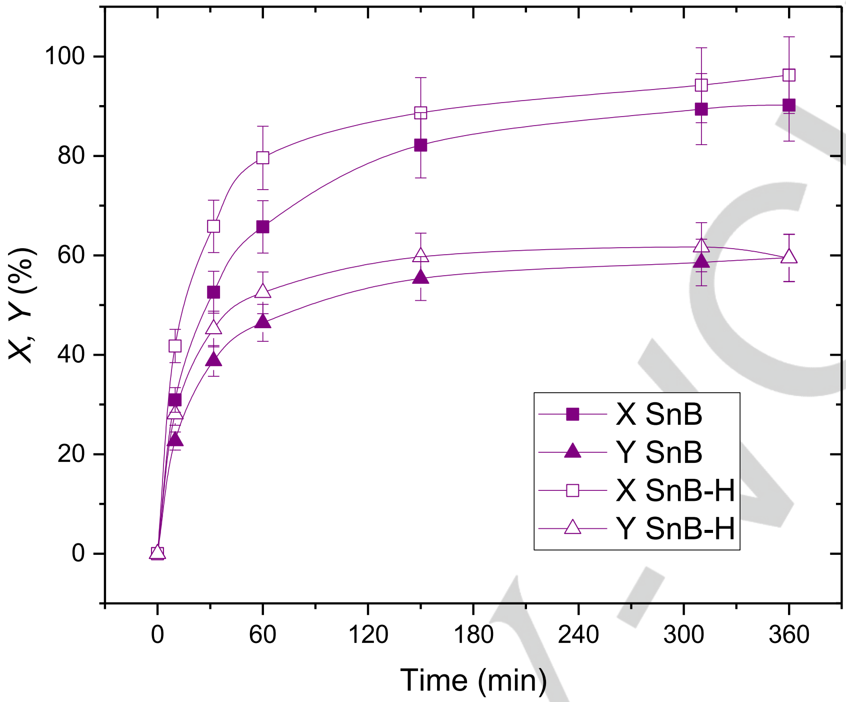

Figure S6. Time online analysis of BVO reaction of 4-Eth-CyO with Sn-β and Sn-β-H, where X represents substrate conversion and Y represents lactone yield.

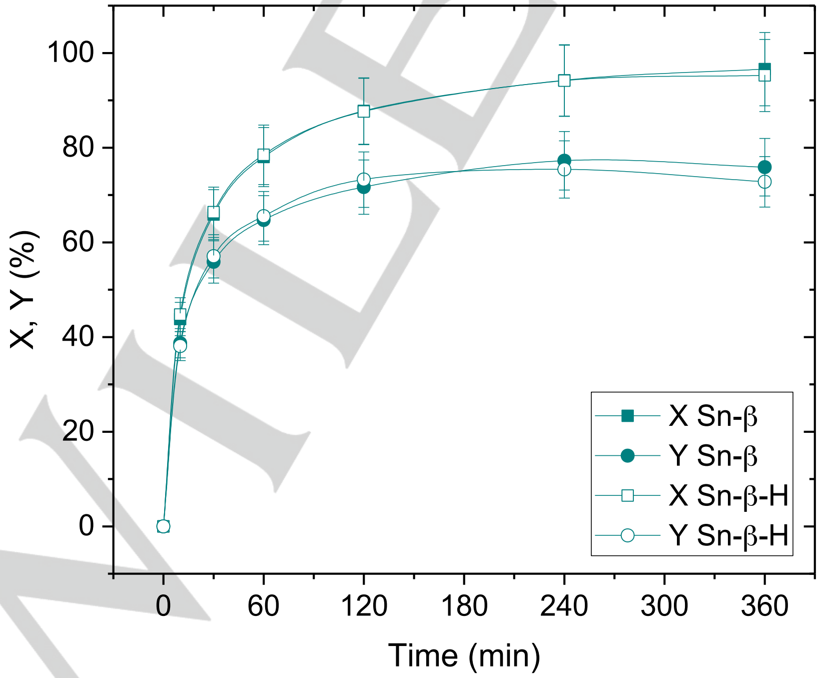

Figure S7. Time online analysis of BVO reaction of 4-^{i}Pr-CyO with Sn-β and Sn-β-H, where X represents substrate conversion and Y represents lactone yield.

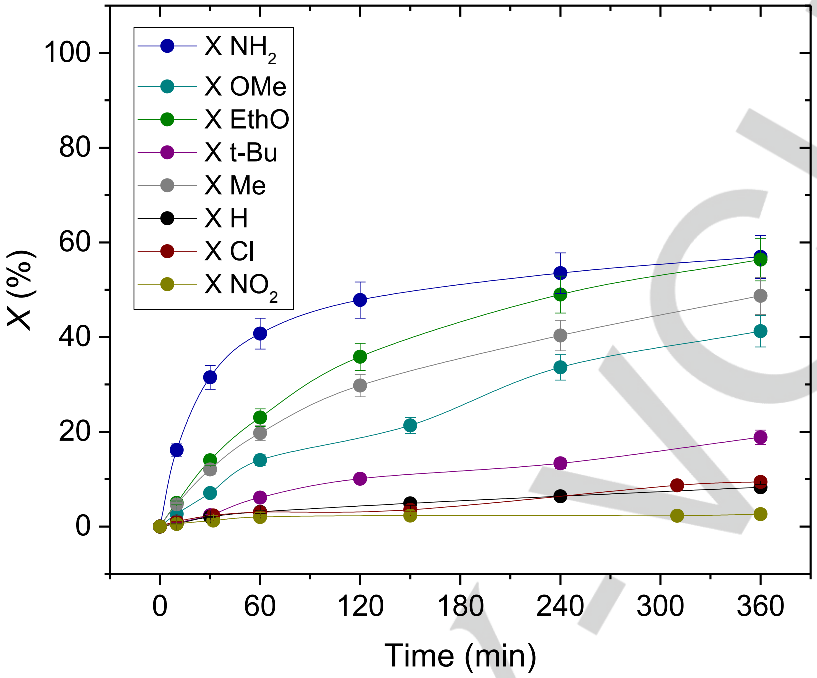

Figure S8. Time online analysis of 4-R-AcetoPh conversion (X) obtained for BVO reaction using Sn-β.

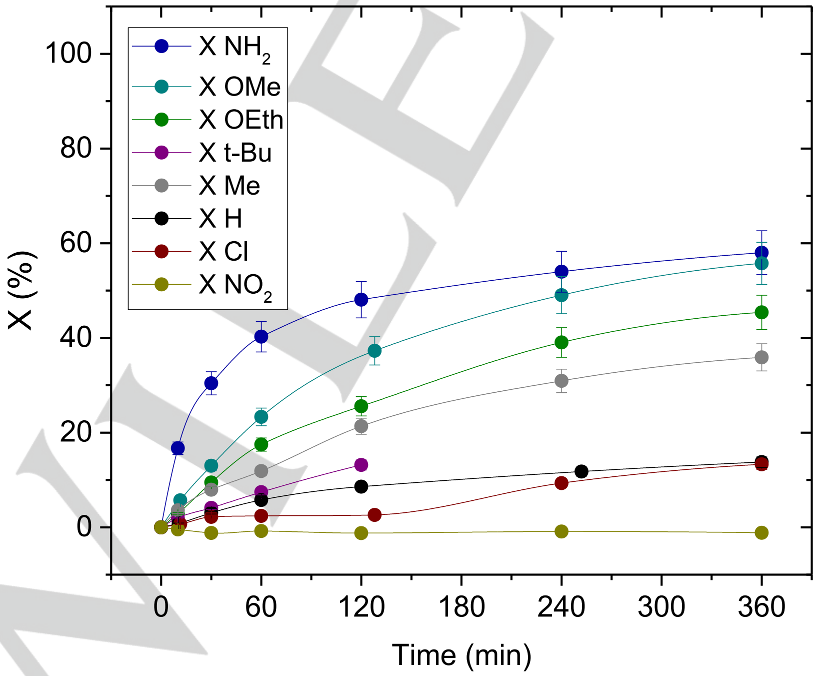

Figure S9. Time online analysis of 4-R-AcetoPh conversion (X) obtained for BVO reaction using Sn-β-H.

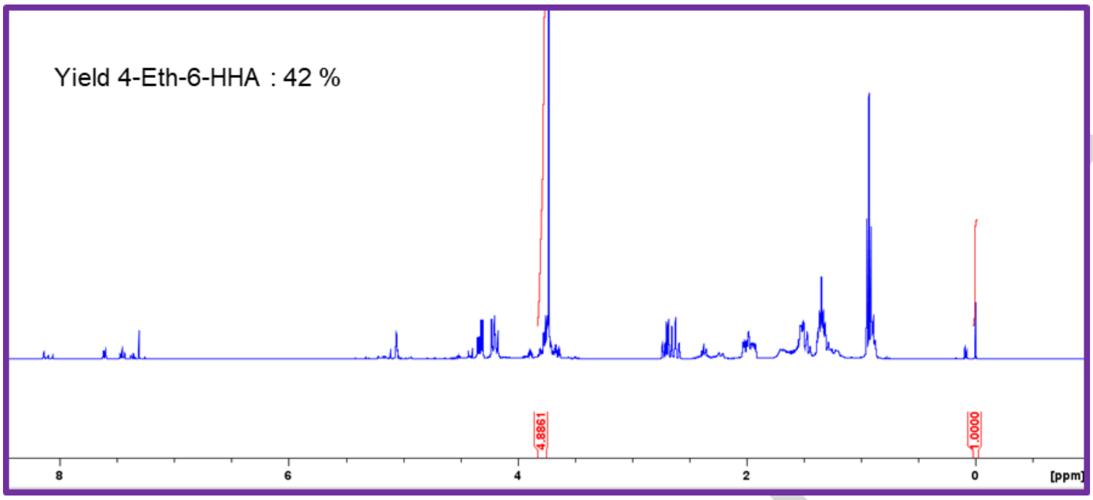

Figure S10. ^{1}H-NMR spectrum of 4-Eth-CyO reaction solution after 6h of reaction.

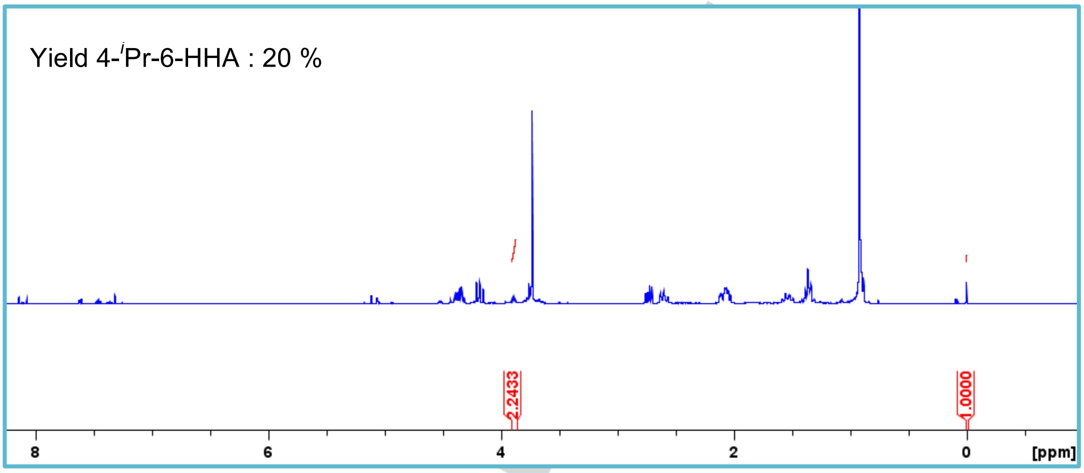

Figure S11. ^{1}H-NMR spectrum of 4-^{i}Pr-CyO reaction solution after 6h of reaction.

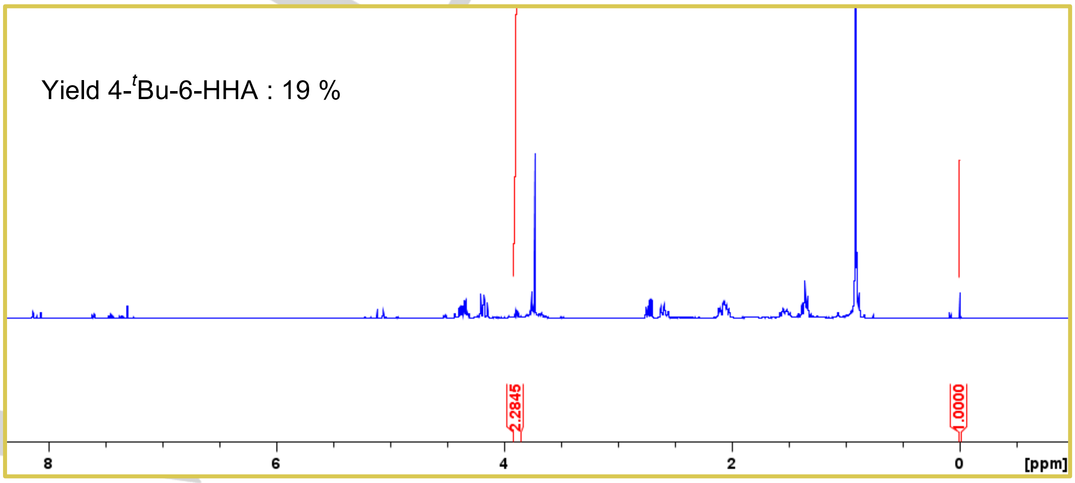

Figure S12. ^{1}H-NMR spectrum of 4-^{t}Bu-CyO reaction solution after 6h of reaction.

Thermodynamics of the hydrolysis of 4-alkyl-caprolactones

The decreased intrinsic reactivity of 4-R-Caprolactones against ε-Caprolactone was also evaluated using Density Functional Theory by calculating the thermodynamics of the hydrolysis reaction for each substrate. Geometry optimisation were performed at the ωB97XD, B3LYP-D3 and M06-2X levels and 398.15 K, using a mixed valence triple ζ basis set 6-311++g(2d,p) for all atoms, and a conductor-like polarisable continuum model of 1,4-dioxane. The results of these calculations are summarised in Table S2. Full coordinates for all the stationary points, together with computed Gibbs free energy and vibrational frequency data, are available via the corresponding Gaussian 09 output files, stored in the digital repository: DOI: 10.6084/m9.figshare.5114035.

Table S2. DFT computed Free Gibbs energies of the hydrolysis of 4-R-caprolactone[a]

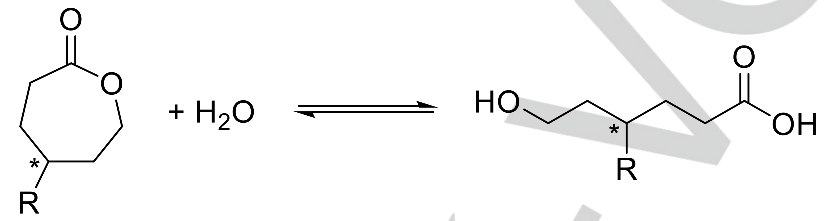

4-R-Caprolactone	ΔG (kcal mol^{-1})[b] ωB97XD functional	ΔG (kcal mol^{-1})[c] B3LYP-D3 functional	ΔG (kcal mol^{-1})[d] M06-2X functional
R = H	+ 4.9	+ 4.5	+ 5.0
R = Me	+ 7.3	+ 6.5	+ 6.3
R = Et	+ 7.9	+ 8.3	+ 8.2
R= ^{i}Pr	+ 7.7	+ 8.4	+ 8.4
R= ^{t}Bu	+ 11.5	+ 7.7	+ 6.7

[a] The smallest value found from both enantiomers (if any) is reported. [b] Protocol: ωB97XD/6-311++g(2d,p)/ 398.15K/cpcm=1,4-dioxane; [c] Protocol: B3LYP-D3/6-311++g(2d,p)/ 398.15K/cpcm=1,4-dioxane; [d] Protocol: M06-2X/6-311++g(2d,p)/ 398.15K/cpcm=1,4-dioxane.
